# The association between dietary, physical activity and the DNA methylation of PPARGC1A, HLA-DQA1 and ADCY3 in pregnant women with gestational diabetes mellitus: a nest case-control study

**DOI:** 10.1186/s12884-024-06673-y

**Published:** 2024-07-26

**Authors:** Yan Wang, Jianhua Ren, Biru Luo

**Affiliations:** 1 Department of Reproductive Medicine Nursing, West China Second University Hospital, Sichuan University, Chengdu, China; 2 Key Laboratory of Birth Defects and Related Diseases of Women and Children (Sichuan University), Ministry of Education, Chengdu, China; 3 Department of Obstetrics and Gynecology Nursing, West China Second University Hospital, Sichuan University, Chengdu, China; 4 Department of Nursing, West China Second University Hospital, Sichuan University, Chengdu, China

**Keywords:** Gestational diabetes mellitus, DNA methylation, Dietary, Physical activity

## Abstract

**Background:**

Gestational diabetes mellitus (GDM) is associated with DNA methylation and lifestyle. The effects of DNA methylation on GDM, and the interaction between DNA methylation and lifestyle factors are not well elucidated. The objective of this study was to explore the association between GDM, DNA methylation and lifestyle factors.

**Methods:**

A nest case-control design was performed. Sociodemographic data, dietary intake and daily physical activity information of pregnant women were collected. Bisulfate pyrosequencing was used to detect the DNA methylation level of *PPARGC1A*, *HLA-DQA1*, and *ADCY3* genes. The differences of DNA methylation levels between the GDM group and the control group were compared. The correlation between clinical characteristics, dietary, physical activity and DNA methylation level was analyzed.

**Results:**

A total of 253 pregnant women were enrolled, of which, 60 participants (GDM: 30; control: 30) were included in the final analysis. There were no significant differences in DNA methylation levels of six methylated sites between the two groups in this study (*P* > 0.05). Daily intake of potato and poultry were associated with DNA methylation level of the CpG 1 site of the *ADCY3* gene in all participants and the control group (*P* < 0.05). Duration of folic acid intake before pregnancy was correlated with the methylation level of the CpG 1 site of the *ADCY3* gene in all participants (*r* = 0.341, *P* = 0.04) and the control group (*r* = 0.431, *P* = 0.025). Daily oil intake was correlated with the methylation level of CpG 2 (*r* = 0.627, *P* = 0.016) and CpG 3 (*r* = 0.563, *P* = 0.036) of *PPARGC1A* in the GDM group.

**Conclusion:**

The association between the DNA methylation levels and GDM wasn’t validated. There were associations between dietary and DNA methylation in pregnant women. A large-sample-sized and longitudinal study is warranted to further investigate the impacts of lifestyle on DNA methylation.

**Supplementary Information:**

The online version contains supplementary material available at 10.1186/s12884-024-06673-y.

## Background

Gestational diabetes mellitus (GDM) is defined as any degree of impaired glucose tolerance first diagnosed during pregnancy [1], which is a common pregnancy complication. It is estimated that 20.4 million (15.8%) women giving birth live in 2019 had some form of hyperglycemia in pregnancy. Among these pregnant women with hyperglycemia, 83.6% were GDM [2]. GDM is proven to have adverse health outcomes for pregnant women and their children, such as obesity and type 2 diabetes [3, 4]. Thus, early prevention and early intervention for GDM benefit maternal and infant health. The exploration of the development mechanism of GDM surely becomes an important basis for the prevention and intervention of GDM.

Studies have confirmed that GDM forms under the influence of both genetic and environmental factors. In recent years, DNA methylation, a kind of epigenetic modification, has been continuously studied and is found to be correlated with GDM and other pregnancy complications [5]. Studies have shown that DNA methylation levels in sub-omental adipose tissue, placenta tissue, and umbilical cord blood of patients with GDM are significantly changed compared with healthy pregnant women [6, 7]. Additionally, studies have found that the methylation levels of some specific genes in sub-omental adipose tissue are closely related to glucose levels and fasting insulin level in pregnant women, indicating that these genes may be involved in the process of insulin resistance and play a key role in the development of GDM [8]. Some researchers found the correlation between DNA methylation of maternal peripheral blood and GDM by high-throughput sequencing technology and identified several methylated sites that may be related to the occurrence of GDM [9, 10]. Moreover, DNA methylation, a dynamic and reversible process, is affected by environmental factors. A review reported that environmental factors such as age, gender, obesity, physical activity, and work stress can lead to DNA methylation modification in some genes (for example *PPARGC1A*,* TNF*,* FTO*,* LEP*,* KCNQ1*, etc.) that increase the risk of type 2 diabetes [11]. It is reasonable to hypothesize that the formation of GDM may have a similar mechanism.

However, in peripheral blood specimens, the results of studies on the relationship between DNA methylation and GDM were inconsistent [10, 12–14]. First, the association between DNA methylation and GDM varied greatly in previous studies [10, 12, 13]. Second, the detected methylated sites were different in different studies [12, 14]. Third, in the absence of studies on the relationship between DNA methylation and GDM in peripheral blood samples in Asian populations, the DNA methylation levels and specific methylation sites of Asian pregnant women with GDM cannot be compared with those of other races. Furthermore, it is unclear whether exposure to environmental factors such as unhealthy diet or sedentary life during pregnancy affects DNA methylation in the peripheral blood of pregnant women. Furthermore, whether environmental factors interact with DNA methylation leading to GDM also remains unknown.

We used the whole genome bisulfite sequencing (WGBS) technology to detect genome-wide DNA methylation in the whole blood specimen. Six DNA specimens were randomly selected from the GDM and normal glucose tolerance (NGT) group (detailed information see supplementary material). After that, a total of six cytosine- phosphate-guanine (CpG) sites of peroxisome proliferator-activated receptor gamma coactivator 1-alpha (*PPARGC1A)*, human leucocyte antigen-DQ alpha 1*(HLA-DQA1)*, adenylate cyclase 3 *(ADCY3)* genes were screened out based on the WGBS result. Secondly, the research on the three genes and GDM or GDM-related factors can provide a theoretical basis and knowledge basis for the verification experiment of this study. *PPARGC1A* has been confirmed in many studies to be involved in blood glucose regulation and the occurrence and development of gestational diabetes in patients with gestational diabetes [15]. *HLA-DQA1* is a known antigen-coding gene closely related to diabetes mellitus [16]. *ADCY3* plays an important role in the regulation of obesity and glucose homeostasis. Several studies and meta-analyses based on Genome wide association study (GWAS) have shown that *ADCY3* gene is associated with overweight/obesity and body mass index [17, 18].

Age, diet, physical activity, obesity, and other environmental factors can affect DNA methylation modification, thus affecting the occurrence and development of GDM. Therefore, we designed a nest case-control study to examine the difference in methylation level of these six CpG sites between GDM and NGT women and to explore whether environmental factors interact with DNA methylation leading to GDM. The aims of this study were: (i) to verify the methylation levels of three differentially methylated genes *PPARGC1A*, *HLA-DQA1* and *ADCY3* in peripheral blood specimens of pregnant women with GDM; (ii) to explore the correlation between dietary intake, physical activity and the DNA methylation level of the above genes.

## Methods

### Study design

A nest case-control study was performed.

### Participants and setting

A study cohort was established between May and December 2019 in West China Second University Hospital of Sichuan University located in southwest China. Participants in this cohort were recruited from the Department of Obstetrics in this hospital using a convenience sampling method. The inclusion criteria of participants were: (1) the age was between 18 and 40 years old; (2) the gestational age was between 18 and 26 weeks; (3) having no serious medical and surgical complications such as heart disease or hypertension; (4) having no pregnancy complications, such as gestational hypertension, placenta previa, etc.; (5) having no mental illness. The participants who had a history of diabetes were excluded.

### Grouping and matching

All participants accepted a one-step 75g oral glucose tolerance test (OGTT) at 24–28 gestational weeks, and GDM was diagnosed according to International Association of Diabetes Pregnancy Study Groups criteria. Then, all participants were divided into the GDM group and the control group according to OGTT results. The GDM group and the control group were matched at a 1:1 ratio. The matching principles were age (± 2 years), gestational age at blood collection (± 2 weeks), and same number of pregnancies (0).

### Sample size

Case-control sample size was calculated using Power Analysis and Sample Size software 2015 (NCSS, USA). DNA methylation levels were regarded as outcome indicators. β value was set to 0.10, α value was set to 0.01, and the estimated shedding rating was 30%. The sample size was calculated as 56 (n_1_ = n_2_ = 28) according to the pilot study results (µ_GDM_ = 0.73, µ_control_ = 0.42, σ_GDM_ = 0.174, σ_control_ = 0.173). The incidence of GDM was estimated to be 17.5% [19]. The study sample size was finally calculated to be 160.

### Data collection

The data were collected on the day when participants entered the study through a face-to-face interview in the outpatient department. A self-designed questionnaire to collect sociodemographic characteristics and clinical information such as age, educational level, religion, ethnicity, occupation, present illness, history of GDM, family history of diabetes, gravidity, parity, history of cesarean section and the number of abortions, etc. The questionnaire took about 30 min to complete.

Data for dietary intake was evaluated using Food Frequency Questionnaire (FFQ), a Chinese version questionnaire amended by Jing [20]. FFQ recorded the frequency and intake amount of thirteen food (rice noodles, cereal, potatoes food, vegetables, fruits, livestock meat, poultry meat, seafood, eggs, milk and dairy products, legumes and soy products, nuts and oils) of participants within the most recent month. Intake of thirteen kinds of food were divided into ten categories (grains, vegetables, fruits, meat, seafood, eggs, milk, beans, nuts, and oils) according to instructions in the study of Jing [20]. The Cronbach’s α coefficient of FFQ in Jing’s study was 0.756. The questionnaire was completed by both participants and researcher W.Y.. The Cronbach’s α coefficient of FFQ in our study was 0.739, suggesting acceptable internal consistency.

Data for physical activity was evaluated using Physical Activity Scale (PAS) [21], which was developed by Aadahl [22], then translated and introduced to China by Jiang [21]. This questionnaire, including 9 items was used in Chinese pregnant women to collect their physical activity regarding the intensity, time spent, and energy expenditure of various categories. The physical activity intensity was classified into 9 categories from A to I, with metabolic equivalent (MET) values of 0.9, 1,1.5, 2.0, 3.0, 4, 5, 6, > 6 METs (1MET = 3.5 ml/ (kg. min)). The reliability and validity of the PAS were confirmed in a previous study [21]. The Cronbach’s α coefficient of PAS was 0.718, suggesting good internal consistency.

Laboratory data were collected on three time points: first trimester of pregnancy (10–13 weeks); second trimester of pregnancy (24–28 weeks); third trimester of pregnancy (32–36 weeks). Biochemical indicators such as hemoglobin level, serum alanine transaminase (ALT) level, serum aspartic transaminase (AST) level, serum γ-glutamyl transpeptidase (γ-GT) level, serum creatinine levels, total cholesterol, high-density lipoprotein (HDL), low-density lipoprotein (LDL) and triglycerides were collected. Glucose metabolism indicators such as fasting plasma glucose, OGTT1h plasma glucose level and 2 h plasma glucose level were also collected.

### Blood collection

The whole blood specimens (2 ml) were collected on the same day as the OGTT test. Specimens were gathered using Ethylene Diamine Tetraacetic Acid (EDTA)-treated tubes and then were stored at -80 °C until nucleic acid extraction.

### DNA extraction and bisulfite treatment

DNA were extracted by DNeasy Blood & Tissue Kit (QIAGEN 69,506, Germany) following manufacturer’s protocol. Nandrop (Thermo, USA) was used to detect the concentration and purity of DNA. The DNA concentration was between 19.8 and 718.9 ng/ µL and the OD260/OD280 ratio was between 1.68 and 1.94, suggesting the high quality of DNA. Bisulfite treatment was performed with the QiagenEpiTect Bisulfite Kit (Qiagen 59,104, Germany) following manufacturer’s protocol. The method of WGBS is displayed in Supplementary material.

### Primer design and polymerase chain reaction (PCR) treatment

PyroMark Assay Design 2.0 software (Qiagen) was used to design gene-specific PCR and sequencing primers (Table 1). The PCR reaction mixture (50uL) was as follow: H_2_O (34.8uL), 5xbuffer GC (KAPA, 10uL), dTNP (10mM/each,1uL), primer (up 50pM/uL,1uL), primer (down 50pM/uL, 1uL), template (2uL), taq (5U/uL, 0.2uL). Condition of the reaction was performed as follow: denaturation at 95˚C for 3 min, 40 cycles of denaturation at 94 °C for 30 s, annealing at 56˚C for 30 s, and extension at 72 °C for 1 min and 72 °C for 7 min.

**Table 1:** Gene-specific PCR and sequencing primers

Chromosome	Detection sequence	Primer	Sequence (5’ to 3’)	
Chr4:23762291–23,762,640 Chr6:32634691–32,634,954 Chr2:24920373–24,920,491	**CG**AGGAATGTAATTGTCATGAGTATTTCCTC TTTATTTTGTTAAGAACTTATT**CG**TGCA**CG**T ATACAATTATATTAAGAAAAGTCTTCATTTT ATTTCCTTTCTTTTGCCTTTATCATGTGACA TAAGATTTATTGACTTCATATCAACG CGTGACCCTGGGAAAAGTCCTTCAACTCTCTGGGCTTTAATGTCCTCCTCGGAAAATGAGAA**CG** GG**CG**CCTTTTCAGACAGAGTGTGAGGGAA**CG**GT	PPARGC1A-1 F(142 bp) PPARGC1A-1R PPARGC1A-1 S PPARGC1A-2 F(142 bp) PPARGC1A-2R PPARGC1A-2 S HLA-DQA1-F(91 bp) HLA-DQA1-R HLA-DQA1-S ADCY3-F(144 bp) ADCY3-R ADCY3-S		AGGTAAAAGAAAGGAAATAAAATGAAGATT CCAACTATAACCACATAACCAATTACA GAAATATTTATGATAATTATATTTT AGGTAAAAGAAAGGAAATAAAATGAAGATT CCAACTATAACCACATAACCAATTACA ATAAAATGAAGATTTTTTTTAATAT AAGTTTTTTAATTTTTTGGGTTTTAATGT AATTCCTAAACTCTTTACATATACTTATCA AATGTTTTTTTAGGAAAATGAGAA AATTAAAGTTTTGAGAAGTATGGGTTGAAG CTTCCAAAACCTCAAACATCAC AGTATGGGTTGAAGG

Note: PCR = polymerase chain reaction; Chr = chromosome; PPARGC1A = peroxisome proliferator-activated receptor gamma coactivator 1-alpha; HLA-DQA1 = human leucocyte antigen-DQ alpha 1; ADCY3 = adenylate cyclase 3. A = adenylate; C = cytosine; G = guanine; T = thymine. Bp = base pair. The detected CpG sites were **bolded**

### Pyrosequencing

In the WGBS results, the methylation differences of the above three genes were statistically significant and relatively significant, and the methylation level differences were 55.71%, 46.8%, and 21.4%, respectively, which met the screening requirements of candidate difference regions. Therefore, pyrosequencing was used to detect the DNA methylation level of the target gene (*PPARGC1A*,* HLA-DQA1*,* ADCY3*). Bisulfite pyrosequencing was performed on a PyroMark Q96 ID (Qiagen) pyrosequencing system. 40 uL PCR product was mixed, soaked, denaturated, and annealed and then reacted with substrate mixture, enzyme mixture, and four dNTP in the detector. The weak light device and the processing software in PyroMark Q96 ID (Qiagen) captured and analyzed the specific detection peak formed by the synthetic visible light during the sequencing reaction to achieve the purpose of real-time DNA sequence determination.

### Statistical analysis

SPSS version 23 was used to analyze data in this study. Frequency and percentage were used to describe qualitative data. Shapiro-Wilk was used to perform the normality test. Mean and standard deviation (*SD*) were used to describe quantitative data of normal or skewed normal distribution. Median and interquartile range (*IQR*) were used to describe quantitative data of non-normal distribution. Categorical data were compared using the Chi-square test. Differences of sociodemographic characteristics, clinical data, and the DNA methylation levels of *PPARGC1A*, *HLA-DQA1*, *ADCY3* genes between groups were assessed by using the independent t-test or non-parametric test. Due to the small number of subjects in each group, physical activity was classified into three groups including resting activity (≤ 1.5METs), light-intensity physical activity (1.6METs-2.9METs), moderate-vigorous intensity physical activity (≥ 3METs) according to the code and MET values of physical activity [23] and the PAS. Spearman or Pearson analysis were used to analyze the correlation between DNA methylation levels of the three genes and dietary intake, physical activity, and biochemical indicators. A *p*-value below 0.05 was considered statistically significant.

## Results

### Socio-demographic and clinical characteristics of participants

**Fig. 1:**
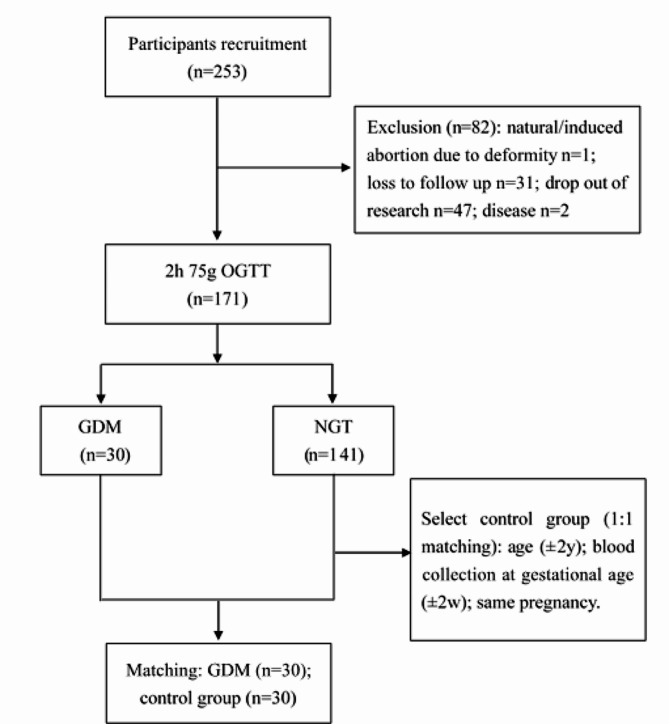
The selection process of participants. OGTT = oral glucose tolerance test; GDM = gestational diabetes mellitus; NGT = normal glucose tolerance; y = year; w = week

After matching, GDM group (*n* = 30) and control group (*n* = 30) were obtained. The selection process of participants is shown in Fig. 1. Table 2 displays the detailed socio-demographic data of participants. The participants of the GDM group had a higher pre-pregnancy body mass index (BMI) (GDM = 22.30 vs. control = 20.35, *t*=-2.464, *P* = 0.017) than that of the control group. The detailed overview of the participants’ clinical characteristics is shown in Supplementary Table 1. The participants of the GDM group had more gravidities than that of the control group (GDM = 2.73 vs. control = 2.00, *t*=-2.552, *P* = 0.013). The GDM group had higher serum creatinine levels in the second trimester (GDM = 40.90 vs. control = 44.83, *t* = 2.402, *P* = 0.031) and plasma prealbumin levels in the third trimester (GDM = 235.52 vs. control = 262.93, *t* = 2.933, *P* = 0.005) than those of the control group. Moreover, OGTT fasting (*t*=-3.933, *P* < 0.001), 1 h (*t*=-8.155, *P* < 0.001), and 2 h (*t*=-9.154, *P* < 0.001) plasma glucose level in the GDM group were higher than those in the control group.

**Table 2:** The demographic of participants

	GDM group (*n* = 30)	control group (*n* = 30)	*t*	*P* value
Age (*M ± SD*, year) Pre-pregnancy weight (*M ± SD*, kg) Height (*M ± SD*, cm) Pre-pregnancy BMI (*M ± SD*, kg/m^2^)	32.47 *±* 3.73 56.13 *±* 9.41 158.60 *±* 4.52 22.30 *±* 3.60	31.93 ± 3.41 52.72 ± 6.87 160.93 ± 5.75 20.35 ± 2.42	-0.578 -1.605 1.748 -2.464	0.566 0.114 0.086 0.017
	N (%)	N (%)	*χ* ^*2*^	*P* value
Ethnicity			-	1.000
Han ethnicity	29 (96.7)	30 (100)		
Minority	1 (3.3)	0 (0)		
Educational level ^*^			0.873	0.350
College degree and below	13 (48.1)	10 (35.7)		
Bachelor or above	14 (51.9)	18 (64.3)		
Religious faith			-	1.000
Yes	1 (3.6)	0 (0)		
No	29 (96.4)	30 (100)		

Note: ^*^ The missing data were not included in the statistical analysis (*n* = 5). GDM = gestational diabetes mellitus; *M* = mean; *SD* = standard deviation; BMI = body mass index.

### **DNA methylation of*PPARGC1A***,*** HLA-DQA1*and*ADCY3***

The detailed information of DNA methylation of *PPARGC1A*,* HLA-DQA1*, and *ADCY3* genes between groups is presented in Table 3. There was no difference in the DNA methylation level of the targeted cytosine-phosphate-guanine (CpG) sites between the GDM group and the control group. The mean methylation level of the *PPARGC1A* gene was similar in the GDM group and the control group (35.72% and 33.60%, respectively), and the mean methylation level of the *ADCY3* gene was also similar in the two groups (respectively 42.87% and 43.58%). The DNA methylation of the CpG site of *HLA-DQA1* had three statuses: full methylated (*n* = 17); 50% methylated (*n* = 22); unmethylated (*n* = 21).

**Table 3:** The DNA methylation level of *PPARGC1A*, *HLA-DQA1*, and *ADCY3*

	GDM group (*n* = 30)	control group (*n* = 30)	t/Z	*P* value
PPARGC1A				
CpG 1 (*M* ± *SD*, %*)*	51.23 ± 5.36	49.75 ± 5.77	1.031	0.307
CpG 2^*^	27.66 (15.09)	21.67 (21.29)	-0.325	0.745
CpG 3 (*M* ± *SD*, %*)*	26.75 ± 11.39	24.50 ± 8.71	0.861	0.393
Mean methylation level (*M* ± *SD*, %*)*	35.72 ± 10.06	33.60 ± 8.71	-0.873	0.387
HLA-DQA1				
CpG 1^*^	56.03 (92.68)	56.32 (90.10)	-0.121	0.904
ADCY3				
CpG 1(*M* ± *SD*, %*)*	55.58 ± 3.23	56.30 ± 2.57	-0.954	0.344
CpG 2 (*M* ± *SD*, %*)*	30.16 ± 2.90	30.86 ± 2.38	-1.019	0.312
Mean methylation level (*M* ± *SD*, %*)*	42.87 ± 2.83	43.58 ± 2.23	1.078	0.285
	N (%)	N (%)	*χ* ^*2*^	*P* value
HLA-DQA1 methylation status			0.106	0.948
100% methylation	9 (30.0)	8 (26.7)		
50% methylation	11 (36.7)	11 (36.7)		
0% methylation	10 (33.3)	11 (36.7)		

Note: ^*^ described as *median* (*IQR)*. GDM = gestational diabetes mellitus; *M* = mean; *SD* = standard deviation; *IQR* = interquartile range; CpG = cytosine-phosphate-guanine.

### Participants’ dietary intake and physical activity

Dietary intake and physical activity of each participant were surveyed (Supplementary Table 2). Fewer participants took folic acid before pregnancy (GDM: 13, control: 15) and most participants took folic acid during pregnancy (GDM: 25, control: 25). The pregnant women in the GDM group ate less beans (GDM = 16 vs. control = 58, *Z*=-2.252, *P* = 0.024) and less oils (GDM = 25.32 vs. control = 30.89, *t* = 2.211, *P* = 0.031) than the pregnant women in the control group. Physical activity was similar in the two groups. The physical activity of participants in the GDM group and the control group was dominated by resting physical activity, followed by light and moderate physical activity. The total activity energy expenditure was 34.30 kcal in the GDM group and 35.51 kcal in the control group (*t* = 0.825, *P* = 0.413).

### Correlation analysis

To explore the association between environmental exposures and DNA methylation, a correlation analysis was performed. Tables 4 and 5, and 6 show the correlation between the DNA methylation level of *PPARGC1A*, *HLA-DQA1*, and *ADCY3* genes and study indicators, respectively. Daily oil intake was correlated with the methylation level of CpG 2 (*r* = 0.627, *P* = 0.016) and CpG 3 (*r* = 0.563, *P* = 0.036) of *PPARGC1A* in the GDM group. Triglyceride in the third trimester was associated with the DNA methylation level of the *HLA-DQA1* gene in the control group (*r*=-0.594, *P* = 0.004) and the GDM group (*r* = 0.571, *P =* 0.001). Postpartum OGTT 2 h blood glucose level of patients with GDM was correlated to the DNA methylation level of CpG 2 of the *ADCY3* gene (*r*=-0.565, *P* = 0.035). Daily intake of potato and poultry were associated with the DNA methylation level of the CpG 1 site of the *ADCY3* gene in all participants and the control group. Duration of folic acid intake before pregnancy was correlated with the methylation level of the CpG 1 site of the *ADCY3* gene in all participants (*r* = 0.341, *P* = 0.04) and the control group (*r* = 0.431, *P* = 0.025).

**Table 4:** The association between DNA methylation level of *PPARGC1A* and clinical characteristics, dietary intake and physical activity of pregnant women

	All participants (*n* = 60)			GDM group (*n* = 30)			Control group (*n* = 30)		
	CpG 1	CpG 2	CpG 3	CpG 1	CpG 2	CpG 3	CpG 1	CpG 2	CpG 3
Parity Prealbumin (first trimester, mg/L) Prealbumin (third trimester, mg/L) γ-GT (third trimester, U/L) Total cholesterol (third, trimester, mg/dl) HDL (third trimester, mmol/L) Duration of folic acid intake during pregnancy (month) Daily oil intake (g) Resting PA Light-intensity PA Moderate-vigorous intensity PA	0.191 -0.299^*^ 0.006 0.007 0.091 0.050 0.206 0.039 -0.125 0.103 0.036	0.150 -0.254 0.135 0.236 0.136 -0.040 0.088 -0.067 -0.012 -0.105 0.152	0.167 -0.282^*^ 0.021 0.155 0.067 -0.081 0.078 -0.058 0.021 -0.090 0.040	-0.008 -0.503^**^ -0.138 -0.109 -0.020 -0.180 0.237 0.444 -0.121 0.210 -0.186	0.100 -0.381 -0.059 0.162 -0.034 -0.325 0.054 0.627^*^ 0.157 -0.195 -0.147	0.062 -0.428^*^ -0.185 0.184 -0.006 -0.243 0.078 0.563^*^ 0.148 -0.105 -0.190	0.378^*^ -0.042 0.266 0.280 0.425^*^ 0.445^*^ 0.248 -0.201 -0.140 -0.045 0.252	0.193 -0.039 0.403^*^ 0.341 0.419 0.384 0.208 -0.338 -0.218 -0.065 0.267	0.269 -0.056 0.430^*^ 0.369 0.394 0.280 0.181 -0.258 -0.149 -0.091 0.246

Note: ^*^P < 0.05; ^**^P < 0.01. GDM = gestational diabetes mellitus; CpG = cytosine-phosphate-guanine; γ-GT = γ-glutamyl transpeptidase; HDL = high-density lipoprotein. Type B activity = 1 metabolic equivalent. Type C activity = 1.5 metabolic equivalent

**Table 5:** The association between DNA methylation level of *HLA-DQA1* and clinical characteristics, dietary intake and physical activity of pregnant women

	All participants (*n* = 60)	GDM group (*n* = 30)	Control group (*n* = 30)
	CpG 1	CpG 1	CpG 1
Parity	0.138	0.024	0.330
Creatinine (first trimester, mmol/L)	-0.254	0.108	-0.479^*^
Ferritin (second trimester, mg/L)	-0.264^*^	-0.271	-0.245
ALT (second trimester, U/L)	-0.340	-0.281	-0.528
AST (second trimester, U/L)	-0.346	-0.341	-0.392
Total cholesterol (third trimester, mmol/L)	0.280^*^	0.115	-0.229
Triglyceride (third trimester, mmol/L)	0.115	0.571^*^	-0.594^*^
LDL (third trimester, mmol/L)	0.307^*^	0.063	0.356
Duration of folic intake acid during pregnancy (month)	0.001	-0.013	0.046
Daily potato intake (g/d)	-0.177	-0.308	-0.032
Resting PA	0.105	0.086	0.078
Light-intensity PA	-0.019	-0.289	0.247
Moderate-vigorous intensity PA	-0.074	0.110	-0.206

Note: ^*^P < 0.05. GDM = gestational diabetes mellitus; CpG = cytosine-phosphate-guanine; ALT = alanine transaminase; AST = aspartic transaminase; LDL = low-density lipoprotein. Type C activity = 1.5 metabolic equivalent, PA = physical activity

**Table 6:** The association between DNA methylation level of *ADCY3* and clinical characteristics, dietary intake and physical activity of pregnant women

	All participants (*n* = 60)		GDM group (*n* = 30)		Control group (*n* = 30)	
	CpG 1	CpG 2	CpG 1	CpG 2	CpG 1	CpG 2
Gravidity Parity Number of abortions γ-GT (second trimester, mmol/L) HDL (third trimester, mmol/L) Fasting glucose (third trimester, mmol/L) OGTT 2 h glucose (postpartum 42 days) Duration of folic acid intake before pregnancy (month) Daily grains intake (g/d) Daily potato intake (g/d) Daily poultry intake (g/d) Resting PA Light-intensity PA Moderate-vigorous intensity PA	-0.395^*^ -0.273^*^ -0.352^*^ -0.145 0.087 -0.055 - 0.341^*^ -0.208 0.312^*^ 0.397^*^ 0.020 -0.095 0.125	0.214 -0.143 -0.207 -0.030 0.211 0.030 - 0.106 -0.312^*^ 0.143 0.157 0.187 -0.169 -0.025	-0.474^**^ -0.301 -0.448^*^ -0.152 0.054 -0.240 -0.514 0.274 -0.200 0.131 0.266 0.133 -0.107 0.045	-0.066 -0.108 -0.053 -0.135 0.282 -0.091 -0.565^*^ 0.157 -0.317 0.267 0.273 0.109 -0.096 0.026	-0.248 -0.219 -0.209 -0.005 0.031 0.397 - 0.431^*^ -0.240 0.485^**^ 0.493^**^ -0.209 -0.030 0.109	-0.457^*^ -0.177 -0.392^*^ 0.159 0.001 0.424 - 0.058 -0.400^*^ 0.013 0.079 0.183 -0.136 -0.149

Note: ^*^P < 0.05; ^**^P < 0.01.GDM = gestational diabetes mellitus; CpG = cytosine-phosphate-guanine; γ-GT = γ-glutamyl transpeptidase; HDL = high-density lipoprotein; OGTT = oral glucose tolerance test. Type C activity = 1.5 metabolic equivalent. Type G activity = 5 metabolic equivalent, PA = physical activity

## Discussion

In this study, no evidence was found to support that the DNA methylation levels of these three genes were related to the occurrence and development of GDM. The DNA methylation level of the most of methylated sites covered in this study was related to environmental exposures including internal and external environmental factors, such as triglyceride, prealbumin, HDL, daily oil intake, duration of folic acid intake, and daily potato intake, etc. These findings suggest that DNA methylation levels in pregnant women appear to be related to factors such as diet, but further studies with larger samples are needed to confirm these results.

In this study, the association between the DNA methylation level of the *PPARGC1A* gene and GDM was not identified. The mean methylation level of the *PPARGC1A* gene in GDM group and control group were (35.72 ± 10.06) % and (33.60 ± 8.71) %, respectively. This result was similar to the result of Qian’s study (GDM: (35.12 ± 6.28) %; non-GDM: (38.76 ± 6.12) %) [8],which was based on sub-omental adipose tissue. It suggested that the methylation levels of the *PPARGC1A* gene in different tissues of different individuals were similar in the whole pregnant women population. Many studies confirmed that the hypermethylated promoter region of the *PPARGC1A* gene may induce the formation of GDM by influencing blood glucose levels [24, 25]. Xie [24] found that the DNA methylation level of -841 site, -810 site, and -216 site in the promoter region of this gene in placenta tissue was positively correlated with the OGTT fasting, 1 h, and 2 h blood glucose level of pregnant women and this association was more significant in women with GDM. A study using pancreatic islet tissue samples presented that hypermethylation of the *PPARGC1A* gene may contribute to type 2 diabetes by lowering mRNA expression levels and then inhibiting glucose-mediated insulin incretion [26], which provides a new direction for the exploration of the pathogenesis of GDM. Similarly, there was no difference in the DNA methylation of *HLA-DQA1* in the GDM group and control group. However, previous studies have confirmed that *HLA-DQA1* is a susceptibility gene for type 2 diabetes [27, 28] and type 1 diabetes [29]. Some studies suggested GDM has HLA genetic background, and HLA-DQA1*0301 [30], *0101 and *0201 [31] allele is a susceptibility gene for GDM patients. In this study, researchers found that the CpG sites of the *HLA-DQA1* gene had three statuses. It was speculated that the reason for this phenomenon may be the existence of single nucleotide polymorphism (SNP), which may impact the methylation level of this gene. Thus, the SNP of the *HLA-DQA1* gene needs to be detected in the future to explore the association between GDM, SNP and the methylation level of the *HLA-DQA1* gene. There was also no association between the methylation level of the *ADCY3* gene and GDM found in this study. *ADCY3* encodes an adenylate cyclase, which catalyzes the synthesis of cyclic adenosine phosphate and plays a crucial role in energy metabolism. Genome-wide Association Studies (GWASs) found that the SNP of *ADCY3* gene was associated with overweight/obesity in European [32], east Asians [33], and Chinese [34] populations. Some studies suggested loss-of-function variants in *ADCY3* increased the risk of obesity and type 2 diabetes [35, 36]. Furthermore, epigenetics studies indicated that the hypermethylated cg17644208 site of *ADCY3* was associated with high BMI [37], and hypermethylation *ADCY3* gene may be related to the existence of proximal SNPs of the *ADCY3* gene in obese patients. Because obesity is a risk factor for t GDM, we speculated that hypermethylation may lead to dysfunction of this gene, thus leading to the development of GDM. There are few studies on the relationship between the DNA methylation of the *ADCY3* gene and GDM. In this study, the correlation between *ADCY3* methylation and GDM was preliminarily explored, the results above indicated that the study on the activity and protein level of this gene was conducive to further understanding of the role of *ADCY3* in GDM. Additionally, the DNA methylation level of CpG 2 of ADCY3 was negatively related to the OGTT 2 h blood glucose level postpartum 42 days. Studies have shown that the upregulation of *ADCY3* gene expression is negatively correlated with the reduction of fasting blood glucose levels and *ADCY3* plays a role in the regulation of glucose homeostasis via insulin secretion [38, 39]. Thus, the association between the DNA methylation level of CpG 2 of *ADCY3* and its gene expression should be further verified to determine whether this methylation site can be used as a potential marker to predict the prognosis of GDM. The reasons for the difference between the results of this study and other studies were as follows: first, selected specimens’ type and methylated sites were different; second, the diagnostic criteria of GDM were different; third, the DNA methylation level was affected by SNP of these three genes. And one reason why no difference was found in this study may be due to the insufficient test efficiency, which needs to be further studied by increasing the sample size.

In this study, we found dietary patterns such as intake of oil, grains, potatoes, and poultry were associated with the DNA methylation level of these three genes. These findings were consistent with previous studies. A recent review synthesized evidence and suggested that dietary factors were closely related to DNA methylation [40]. Some studies found that leukocyte LINE-1 methylation level, a surrogate marker of global DNA methylation in peripheral blood was associated with dietary patterns in women and cancer-free populations [41, 42]. In this study, duration of folic acid intake before pregnancy was related to the DNA methylation level of P. 1 site of the *ADCY3* gene. Folate provides a single-carbon component of the S-adenosylmethionine synthesis pathway. S-adenosylmethionine is a major cellular methyl donor that affects methylation reactions [43]. Folic acid supplementation provides humans with folate and alters global genome DNA methylation profiles [44]. Many studies based on animal models and human populations confirmed that supplementation with folic acid decreased insulin resistance, induced DNA methylation in genes associated with obesity and insulin secretion, and improved blood glucose control in obese patients with type 2 diabetes [45–47]. Based on the evidence above, it can be hypothesized that folic acid supplementation may affect the methylation of the *ADCY3* gene, thereby affecting the function of this gene and leading to obesity. A prospective large sample study could be used for validation in the future. Additionally, the association between daily intake of oil, grains, potatoes, and poultry meat and the DNA methylation level of some CpG sites of *PPARGC1A*, *HLA-DQA1*, and *ADCY3* genes was also observed. Due to the lack of studies on the relationship between methylation of these three genes and diet, the results of this study cannot be supported by other studies. However, a recent study found that intake of whole-meal bread and potatoes positively correlated with LINE-1 methylation levels, and vegetable oil negatively correlated with LINE-1 methylation levels [41]. Prudent dietary pattern (characterized by a high intake of vegetables and fruits) had a higher average LINE-1 methylation level than Western dietary patterns (characterized by a high intake of meats, grains, dairy, oils, and potatoes) [41, 42]. It was suggested that the dietary patterns of participants could be divided into prudent and western groups for further study.

The association between physical activity and DNA methylation level of *PPARGC1A*, *HLA-DQA1* and *ADCY3* were also explored in this study, and no statistical difference was found. Some studies confirmed that physical activity can alter the DNA methylation pattern in skeletal muscle, adipose tissue, and peripheral blood [48–50]. A cohort study detected two new methylated CpG sites with a nonlinear dose-response relationship to moderate-vigorous physical activity [51]. A study based on the American cancer-free population showed that f an elevated risk of global hypomethylation associated with low levels of physical activity in non-Hispanics [52]. The biological mechanisms by which physical activity benefits individuals include reducing insulin resistance, fat mass, and inflammation, and altering the metabolism of endogenous steroid hormones [53]. Physical activity is one of the few modifiable behaviors and may affect the risk of GDM through changing DNA methylation. Future studies with a larger sample size and a longitudinal design are warranted to further investigate its impact on DNA methylation.

This study had some limitations. First, only one hospital was included in this study. Thus, this may reduce the representativeness of the sample. Second, False negative cannot be ruled out because the sample size was small, with only 60 cases. Finally, we didn’t consider pre-pregnancy BMI or gestational weight gain, a high-risk factor for GDM as matching factors, which limited the examination for the association between obesity, GDM and the DNA methylation level. A large-sample-sized and longitudinal study is warranted to further examine the association between obesity, GDM, and DNA methylation level and investigate the impacts of lifestyle on DNA methylation.

## Conclusions

In summary, the significant difference of the DNA methylation levels of these six CpG sites of *PPARGC1A*, *HLA-DQA1*, and *ADCY3* genes between the GDM group and the control group was not found. Dietary patterns such as intake of oil, grains, potatoes, etc. were associated with the DNA methylation level of these three genes, suggesting the association between dietary and DNA methylation. However, studies with a larger sample size and a longitudinal design are warranted to further investigate its impact on DNA methylation in the future.

### Electronic supplementary material

Below is the link to the electronic supplementary material.

Supplementary Material 1

Supplementary Material 2

## Data Availability

The datasets generated and/or analyzed during the current study are available in the Science Data Bank repository, 10.57760/sciencedb.11974.

## Abbreviations

GDM: gestational diabetes mellitus
DNA: Deoxyribonucleic acid
PPARGC1A: Peroxisome proliferator-activated receptor gamma, coactivator 1 alpha
HLA-DQA1: Human leucocyte antigen-DQA1
ADCY3: Adenylate cyclase 3
