# Supplementary material for: The association between dietary, physical activity and the DNA methylation of PPARGC1A, HLA-DQA1 and ADCY3 in pregnant women with gestational diabetes mellitus: a nest case-control study
Source: BMC Pregnancy Childbirth. 2024 Jul 26;24:503. doi: 10.1186/s12884-024-06673-y (PMC11282794; doi:10.1186/s12884-024-06673-y)
Supplement: Supplementary file 1 — Supplementary Material 1 [file 12884_2024_6673_MOESM1_ESM.docx]

**Whole genome bisulfate sequencing (WGBS)**

**Library preparation and quantification**

Six DNA specimens were randomly selected from GDM (n=3) and normal glucose tolerance group (n=3) for WGBS. A total amount of 5.2 microgram genomic DNA was fragmented by sonication to 200-300bp with Covaris S220, followed by end repair and adenylation. Then these DNA fragments were treated twice with bisulfite using Accel-NGS® Methyl-Seq DNA Library Kit, before the resulting single-strand DNA fragments were PCR amplificated using KAPA HiFi Hot Start Uracil + Ready Mix (2X). Library concentration was quantified by Qubit® 2.0 Flurometer (Life Technologies, CA, USA) and quantitative PCR, and the insert size was assayed on Agilent Bioanalyzer 2100 system.

**Sequencing and mapping**

The library preparations sequenced at the Novogene Bioinformatics Institute (Beijing, China) on an Illumina HiSeq 2000/2500 platform and 125bp/150bp paired-end reads were generated. Bismark software (version 0.16.3) was used to perform alignments of bisulfite-treated reads to a reference genome. Sequence reads that produce a unique best alignment from the two alignment processes (original top and bottom strand) are then compared to the normal genomic sequence and the methylation state of all cytosine positions in the read is inferred.

**Calculating methylation level**

To identify the methylation site, we modeled the sum Mc of methylated counts as a binomial (Bin) random variable with methylation rate r.

𝐦𝐂~𝐁𝐥𝐧(𝐦𝐂 + 𝐮𝐦𝐂 ∗ 𝐫)

In order to calculate the methylation level of the sequence, we divided the sequence into multiple bins, with bin size is 10kb. The sum of methylated and unmethylated read counts in each window were calculated. Methylation level (ML) for each window or C site shows the fraction of methylated Cs, and is defined as:

***ML*(C)=**$\frac{\boldsymbol{reads}\mathbf{(}\boldsymbol{mC}\mathbf{)}}{\boldsymbol{reads}\left( \boldsymbol{mC} \right)\mathbf{+}\boldsymbol{reads}\mathbf{(}\boldsymbol{C}\mathbf{)}}$

Calculated ML was further corrected with the bisulfite non-conversion rate according to previous studies. Given the bisulfite nonconversion rate r, the corrected ML was estimated as:

$\boldsymbol{ML}_{(corrected)}$=$\frac{\boldsymbol{ML}\boldsymbol{-}\boldsymbol{r}}{\boldsymbol{1}\boldsymbol{-}\boldsymbol{r}}$

**Differentially methylated regions (DMRs) analysis**

Differentially methylated regions (DMRs) were identified using the DSS software. The core of DSS is a new dispersion shrinkage method for estimating the dispersion parameter from Beta-Binomial distributions. DMRs across the whole genome using a sliding window approach. The window size and step were set at 1000 and 100 bp, respectively. Its workflow mainly includes:

i) Estimating the methylated level of site; ii) Distributed evaluation: in this process, all site information is introduced to provide more accurate distribution evaluation; iii) DMR calling: Wald test was conducted for each site to obtain P value; Finally, DMR is determined according to *P* value and some DMR identification conditions. Screening methods: the proportion of sites with P value less than 1e-05 was greater than 50% of the region, the number of regional sites was greater than 3, and the length was greater than 50. And when the distance between two DMR is less than 100bp, these two regions are merged.

DSS possess three characteristics to detect DMRs. First, spatial correlation. Proper utilization of the information from neighboring Cytosine sites can help improve estimation of methylation levels at each Cytosine site, and hence improve DMR detection. Second, the read depth of the Cytosine sites provides information on precision that can be exploited to improve statistical tests for DMR detection. Finally, the variance among biological replicates provides information necessary for a valid statistical test to detect DMRs, when there is no biological replicate, DSS combining data from nearby Cytosine sites and using them as ‘pseudo-replicates’ to estimate biological variance at specific locations.

According to the distribution of DMRs through the genome, we defined the genes related to DMRs as genes whose gene body region (from TSS to TES) or promoter region (upstream 2kb from the TSS) have an overlap with the DMRs.
