# Supplementary material for: The association between dietary, physical activity and the DNA methylation of PPARGC1A, HLA-DQA1 and ADCY3 in pregnant women with gestational diabetes mellitus: a nest case-control study
Source: BMC Pregnancy Childbirth. 2024 Jul 26;24:503. doi: 10.1186/s12884-024-06673-y (PMC11282794; doi:10.1186/s12884-024-06673-y)
Supplement: Supplementary file 2 — Supplementary Material 2 [file 12884_2024_6673_MOESM2_ESM.docx]

Supplementary table 1 The clinical characteristics of participants.

|  | GDM group (n=30) | control group  (n=30) | *t* | *P* value |
| --- | --- | --- | --- | --- |
| Gestational age at blood collection (*M*±SD, week)  Gravidity *(M*±SD, time)  Parity *(M*±SD, time)  Miscarriages *(M*±SD, time)  First trimester (10-13 week)  Prealbumin (*M*±SD, mg/L)  Ferritin (*M*±SD, ug/L)  Creatinine (*M*±SD, umol/L)  ALT (*M*±SD,IU/L)  AST (*M*±SD,IU/L)  γ-GT (*M*±SD,IU/L)  Fasting glucose (*M*±SD, mmol/L)  Second trimester (24-28 week)  Prealbumin (*M*±SD, mg/L)  Ferritin (*M*±SD, ug/L)  Creatinine (*M*±SD, umol/L)  ALT (*M*±SD, IU/L)  AST (*M*±SD, IU/L)  γ-GT (*M*±SD, IU/L)  OGTT fasting (*M*±SD, mmol/L)  OGTT 1hour (*M*±SD, mmol/L)  OGTT 2hour (*M*±SD, mmol/L)  Glycosylated hemoglobin (*M*±SD, %)  Third trimester (32-36 week)  Prealbumin (*M*±SD, mg/L)  Ferritin (*M*±SD, ug/L)  Creatinine (*M*±SD, umol/L)  ALT (*M*±SD, U/L)  AST (*M*±SD, U/L)  γ-GT (U/L)  Fasting glucose (*M*±SD, mmol/L)  Total cholesterol (*M*±SD, mmol/L)  Triglyceride (*M*±SD, mmol/L)  HDL (*M*±SD, mmol/L)  LDL (*M*±SD, mmol/L) | 25.12 ±1.32  2.73 ±1.34  0.53±0.51  1.23±1.17  246.69±35.68  76.33±74.98  42.50±6.31  27.69±19.70  24.46±12.44  20.69±17.75  4.55±0.33  248.86±38.25  37.91±28.94  40.90±4.07  27.00±20.22  27.57±17.49  42.41±58.11  4.52±0.33  10.10±1.29  8.71±1.01  4.74±0.41  235.52±38.91  29.51±22.44  43.33±5.46  28.50±25.42  25.54±13.08  27.07±44.09  4.84±1.15  5.95±1.29  3.50±1.28  1.80±0.47  3.31±1.42 | 24.71±1.14  2.00±0.83  0.30±0.47  0.73±0.74  236.92±27.85  76.89±49.61  43.37±5.5  25.47±18.51  22.95±7.76  22.27±16.01  4.37±0.52  242.18±32.44  37.94±24.34  44.83±2.48  23.09±14.34  21.73±5.41  16.09±9.96  4.19±0.32  7.27±1.40  6.19±1.11  4.69±0.24  262.93±29.97  25.50±17.90  42.98±7.97  18.71±8.5  19.89±4.85  18.71±14.93  4.52±0.93  6.24±0.78  3.79±1.61  2.20±0.42  3.26±0.50 | -1.290  -2.552  -1.855  -1.984  -1.094  0.032  0.447  -0.424  -0.533  0.336  -1.431  -0.462  0.004  2.402  -0.542  -1.065  -1.479  -3.933  -8.155  -9.154  -0.362  2.933  -0.725  -0.190  -1.932  -2.140  -0.950  1.077  -0.613  -0.445  -2.044  0.093 | 0.202  0.013  0.069  0.052  0.279  0.975  0.658  0.673  0.596  0.738  0.159  0.648  0.997  0.031  0.593  0.298  0.153  <0.001  <0.001  <0.001  0.719  0.005  0.471  0.850  0.059  0.037  0.346  0.287  0.548  0.662  0.056  0.927 |
|  | N (%) | N (%) | *χ^2^* | *P* value |
| Hepatitis B  Hyperthyroidism  Hypothyroidism  Family history of hypertension  Family history of diabetes  History of GDM  History of cesarean section  Multiple pregnancy  Received IVF-ET | 4 (13.3)  1 (3.3)  4 (13.3)  3 (10.0)  6 (20.0)  3 (10.0)  9 (30.0)  1 (3.4)  5 (16.7) | 1 (3.3)  0 (0)  0 (0)  6 (20.0)  8 (26.7)  0 (0)  6 (20.0)  1 (3.4)  2 (6.7) | 0.873  -  2.411  0.523  0.373  1.404  0.800  0.001  0.647 | 0.350  1.000  0.121  0.470  0.542  0.236  0.371  1.000  0.421 |

Note: *M*=mean; *SD*= standard deviation. GDM=gestational diabetes mellitus; ALT= alanine transaminase; AST=aspartic transaminase; γ-GT=γ-glutamyl transpeptidase; HDL= high-density lipoprotein; LDL=low-density lipoprotein; OGTT=oral glucose tolerance test; IVF-ET= in vitro fertilization and embryo transfer.

Supplementary table 2 The dietary intake and physical activity of participants in the GDM group and the control group.

|  | GDM group (n=30) | | control group (n=30) | *t/Z* | *P* value |
| --- | --- | --- | --- | --- | --- |
|  | *M*±SD*/median (IQR)* | | *M*±SD*/median (IQR)* |  |  |
| **Dietary intake**  Duration of folic acid intake before pregnancy (month)  Duration of folic acid intake during pregnancy (month)  Grains (g/day) ^*^  Vegetables (g/day)  Fruits (g/day)  Meat (g/day)  Seafood (g/day)  Eggs (g/day)  Beans (g/day)  Nuts (g/day)  Milk (g/day)  Oils (g/day) ^*^  Carbohydrate (g/day)  Protein (g/day)  Fat (g/day)  **Physical activity** (h/day)  Resting physical activity  Light-intensity physical activity^*^  Moderate-vigorous intensity physical activity^*^  Total activity energy expenditure (MET*h) ^*^ | 0 (2.75)  5.0 (1.56)  335.08±167.36  300 (437)  300 (250)  161 (175.63)  29 (34.44)  50 (14.50)  16 (60.22)  29 (73)  250 (343.13)  25.32 ±8.41  316.20 (182.36)  95.03 (105.94)  117.42 (141.70)  13.00 (3.78)  7.05±2.71  2.47±1.49  34.30±4.71 | 2.0 (3.0)  3.0 (2.0)  345.43±145.98  350 (400)  300 (287.50)  212.75 (300.83)  30 (61.63)  50 (30.25)  58 (144.50)  17.5 (85.69)  250 (260)  30.89 ±10.92  389.07 (209.26)  142.23 (106.53)  108.79 (104.60)  13.74 (3.35)  7.19±2.10  2.67±1.90  35.51±6.11 | | -0.950  -1.926  0.247  -0.420  -0.025  -0.934  -0.312  -0.795  -2.252  -0.969  -0.279  2.211  -0.655  -1.376  -0.246  -0.591  -0.221  -0.436  0.825 | 0.347  0.054  0.806  0.680  0.984  0.355  0.760  0.433  0.024  0.337  0.786  0.031  0.512  0.169  0.806  0.555  0.826  0.665  0.413 |

Note: ^*^ described as *mean* and *SD.* *M*=mean; *SD*= standard deviation. GDM=gestational diabetes mellitus; *M*=mean; *SD*= standard deviation; *IQR*=interquartile range; PA=physical activity; MET= metabolic equivalent, 1MET=3.5ml/ (kg. min).
